# Time-Frequency Methods for Structural Health Monitoring ^†^

**DOI:** 10.3390/s140305147

**Published:** 2014-03-12

**Authors:** Alexander L. Pyayt, Alexey P. Kozionov, Ilya I. Mokhov, Bernhard Lang, Robert J. Meijer, Valeria V. Krzhizhanovskaya, Peter M. A. Sloot

**Affiliations:** 1 Siemens LLC, Corporate Technology, Volynskiy lane 3A, St. Petersburg, 191186, Russia; E-Mails: alexey.kozionov@siemens.com (A.P.K.); ilya.mokhov@siemens.com (I.I.M.); 2 University of Amsterdam, Science Park 904, 1098 XH, Amsterdam, The Netherlands; E-Mails: V.Krzhizhanovskaya@uva.nl (V.V.K.); P.M.A.Sloot@uva.nl (P.M.A.S.); 3 St. Petersburg State University of Aerospace Instrumentation, Bolshaya Morskaia 67, St. Petersburg, 190000, Russia; 4 Siemens AG, Corporate Technology, Muenchen, 80200, Germany; E-Mail: bernhard.lang@siemens.com; 5 Nederlandse Organisatie voor Toegepast Natuurwetenschappelijk Onderzoek (TNO), Eemsgolaan 3 NL-9727 DW, Groningen, The Netherlands; E-Mail: robert.meijer@tno.nl; 6 National Research University ITMO, St. Petersburg, 197101, Russia; 7 St. Petersburg State Polytechnic University, St. Petersburg, 195251, Russia; 8 Nanyang Technological University, 639798, Singapore

**Keywords:** anomaly detection, structural health monitoring, time-frequency analysis, sensors, flood protection systems, levee monitoring, one-side classification, leakage detection

## Abstract

Detection of early warning signals for the imminent failure of large and complex engineered structures is a daunting challenge with many open research questions. In this paper we report on novel ways to perform Structural Health Monitoring (SHM) of flood protection systems (levees, earthen dikes and concrete dams) using sensor data. We present a robust data-driven anomaly detection method that combines time-frequency feature extraction, using wavelet analysis and phase shift, with one-sided classification techniques to identify the onset of failure anomalies in real-time sensor measurements. The methodology has been successfully tested at three operational levees. We detected a dam leakage in the retaining dam (Germany) and “strange” behaviour of sensors installed in a Boston levee (UK) and a Rhine levee (Germany).

## 1. Introduction

### 1.1. Flood Defence Structures

There are thousands of kilometres of flood defence structures around the World protecting infrastructure and populations against floods. There are about four times more floods registered nowadays as compared to the 1980s [1], some of them caused by weaknesses in flood defence structures.

Flood defences are defined as defences which protect against flooding by a river or the sea [2]. There are different types of flood protection structures. Usually they are classified as fluvial and coastal [2]. Vertical wall, slope (or embankment), high ground and culverts are related to the first class; vertical seawalls, sloping seawalls and beaches are related to the second class.

A *dam* is as an artificial barrier constructed across a watercourse for the purpose of storage, control, or diversion of water [3]. Dams are typically constructed of earth, rock or concrete. Based on structure and design, dams can be classified in the following manner: gravity, embankment, arch, buttress and others [4].

*Embankment dams* are made from compacted earth, and have two main types: *rock-fill* and *earth-fill* dams. Embankment dams rely on their weight to hold back the force of water.

A *levee* is a natural or an artificial structure designed to contain, control, or divert the flow of water to prevent flooding of adjacent lands. Artificial levees are constructed using various materials, e.g., soil, rock, concrete [3].

*Dikes* (or dykes) are typically earth-fill dams [5]. The terms *dike* and *levee* are often used interchangeably. Historically a dike is used to defend against storm surges from the sea such as the system of dikes that protects The Netherlands. A levee stops flood waters from streams and lakes such as the system of levees that protects cities along the Mississippi River [6].

In our work we consider two river dikes and one retaining dam. One of the purposes of retaining structures is to create large bodies of water, or reservoirs that have a variety of functions, including land irrigation, power generation, water supply and flood control [7].

The performance of a defence structure depends [2] on the magnitude of the loads (water level, waves, wind, traffic *etc.*) acting on the structure; response of the structure to the loading; and performance of the foundation (especially important for the embankments).

**Figure 1.**
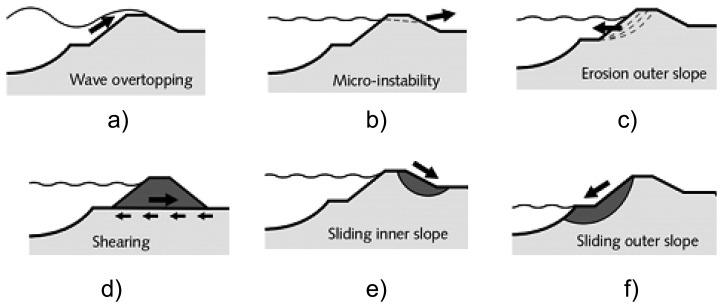
Classification of dam failures [10]: (**a**) wave overtopping; (**b**) micro-instability; (**c**) erosion of the outer slope; (**d**) horizontal shear; (**e**) sliding of the inner slope; (**f**) sliding of the outer slope.

According to a study of dam failures in the USA [8] overtopping (Figure 1a) is the reason for 34% of the observed floods. Foundation defects due to differential settlement, slides, slope instability, uplift pressures, and foundation seepage lead to 30% of all dam failures. Failure due to piping and seepage accounts for 20% of all failures. The remaining 16% of failures are caused by the problems with conduits and valves, and other miscellaneous problems.

Between 1134 and 2006 there were 1,735 dike failures in The Netherlands [9]. Of these events 67% were caused by erosion of inner slope protection, 11% by ice drift, 6% by erosion or instability of outer slope protection (Figure 1c), 5% by sliding inner slopes (Figure 1e), 4% by external reasons (human and animal), 3% by sliding outer slopes (Figure 1f), 2% by liquefaction of the shore line, 1% by piping, 1% by micro-instability (Figure 1b), horizontal shear (Figure 1d) and other related mechanisms.

### 1.2. Dam Health Monitoring

The mechanism of a possible failure is unknown beforehand and is therefore difficult to predict. Visual inspection cannot guarantee detection of the onset of a levee failure early enough to prevent its collapse, therefore a continuous levee health monitoring process is required. Development of physical models could provide a robust solution for levee behaviour assessment [11], but these rarely include real-time health monitoring. For continuous dike monitoring two approaches are used: remote sensing by LiDAR [12] or by satellite [13] and by sensors installed inside the dike. The use of fibre optic cables for deformation analysis is described in [14]. The advantage of the first method is that it is non-intrusive. The second method is more accurate and reliable.

In our research we install sensors into the levees to monitor their condition. Pore water pressure sensors proved to be useful in levee stability analysis [15]. Inclinometers are generally used to measure tilt and to monitor lateral movements for embankments and dams [16]. Leakage can be detected by fibre optic sensors measuring the temperature inside the levee [17]. A detailed overview and comparison of existing sensor technologies for levee monitoring can be found in [18].

Automated generation of early warning alarms using real-time streams of sensor measurements requires dedicated data-driven methods. For instance, the application of singular value decomposition (SVD) to distributed temperature values is suggested for automatic leakage detection in [19]. Artificial neural networks were applied for slope stability analysis in [20].

Modern sensor technologies and intelligent data processing methods have been developed by the *UrbanFlood* project for early detection of anomalies in flood protection systems. In this paper, we present a robust data-driven anomaly detection method that combines time-frequency feature extraction, using wavelet analysis and phase shift (time-frequency feature for monitoring of phase difference between oscillating signals of different sensors) with one-sided classification techniques to identify onset of failure anomalies in real-time. The methodology has been successfully tested at three operational levees. We detected a dam leakage in a retaining dam (Germany), and sensor malfunctions in the Boston levee (UK), and a non-saturated area in a Rhine levee (Germany). This paper includes results previously presented in [21]. In the next Section we introduce the general anomaly detection approach and present the one-side classification method (Neural Clouds) and describe the applied feature extraction methods.

## 2. Anomaly Detection Approach

### 2.1. General Concept

One of the main goals of the *UrbanFlood* project was the development of an on-line early warning system (EWS) based on levee health monitoring [22,23]. Sensor networks were installed into several pilot sites in The Netherlands, Germany and in the United Kingdom. More details about these pilot sites can be found in [24].

Sensor data are processed by the Artificial Intelligence (AI) component—part of the *UrbanFlood* EWS platform. More information about the early warning system can be found in the official site of the project—www.urbanflood.eu. If there are anomalies in sensor measurements due to different triggers (e.g., sensor fault or real developing failure), the AI component triggers an alarm. The most important benefit of this approach is that it uses reference data related to normal behaviour of the monitored object. This is possible due to a one-side classification approach using Neural Clouds (NC) [25].

**Figure 2.**
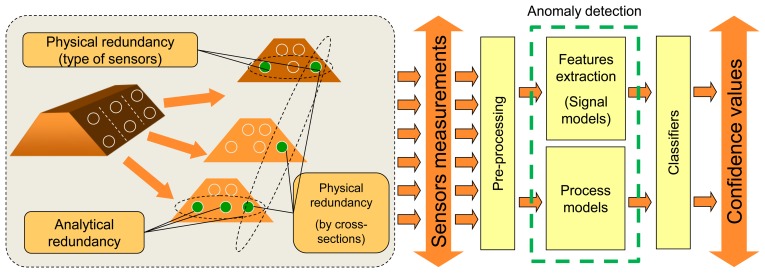
Anomaly detection workflow.

Multidimensional sensor measurements can be grouped according to so-called physical redundancy (see Figure 2), whereby signals from neighbour sensors, sensors from the same cross-section or sensors measuring the same physical parameters can be analyzed by one classifier. Another way of grouping is evaluation of analytical redundancy between the sensors—sensors are grouped if a clear and stable relation between sensor values is detected. In this way the consistency of detected dependencies is monitored.

Pre-processing is usually required due to different problems with the raw data. For example, multidimensional data is usually not synchronised in time due to serial polling of individual sensors. This means that alignment of measurements to the common time grid is required. This can be done by a simple linear interpolation or by application of more sophisticated but well known algorithms. In our work we applied Singular Spectrum Analysis (SSA); the details will be published in [26].

Time-frequency (Short-Time Fourier Transformation - STFT) and frequency methods (Fast Fourier Transformation - FFT) have been applied at the feature extraction stage for the Zeeland dike data analysis [27], time-frequency methods (wavelet transformation) have been used for Rhine levee data analysis [21]. Process models were used for the Boston levee data analysis [28]: feed-forward neural networks and autoregressive models with exogenous inputs (ARX) were constructed for sensor fault detection. In this work we combine two approaches: feature extraction (wavelet transformation) and process models (analysis of the phase shift based on FFT). These metrics are described later in this Section.

In the next subsections we describe the two most important steps of the workflow: feature extraction and classification.

### 2.2. Time-Frequency Feature Extraction Methods

#### 2.2.1. Wavelet Analysis

Leakage is the main issue for earthen and concrete dams [29]. Comprehensive analysis of various methods for leakage detection can be found in the [30]. Analysis of relationship between reservoir level and flow rate is one of the possible ways for the detection of a developing failure. Various dike failure scenarios due to leakage are described in [31].

We can represent leakage as two rapid changes in a spatial set of observations: much lower temperatures in the area of leakage [32]. There are different approaches to detect such rapid changes in time series, e.g., Student's T-test [33]. A detailed overview of more advanced detection methods is presented in [34]. Zhang [35] uses wavelets for abrupt fault detection. Percival [36] applied wavelets to analyse the water temperature measurements from the Wivenhoe Dam. Each signal was decomposed using wavelets into daily, sub-annual and annual (DSA) components. Each of the components was used for further analysis.

**Figure 3.**
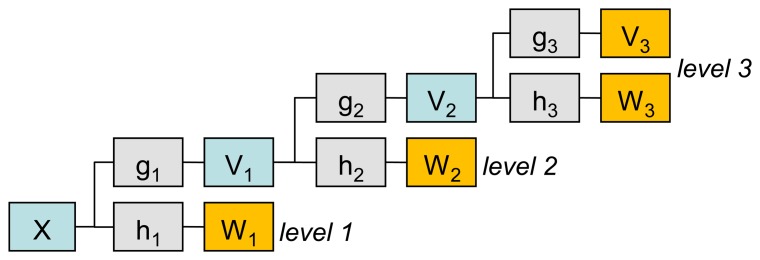
MODWT cascade algorithm. *X* is the analysed signal, *g_i_* and *h_i_* are scaling (low-pass) and wavelet (high-pass) filters respectively, *v_i_* and *w_i_* are approximation and detail MODWT coefficients of the *i*-th level of decomposition respectively.

We choose the Maximum Overlap Discrete Wavelet Transform (MODWT) [37] for feature selection. MODWT is a computationally efficient method for time-frequency representation of time series. The MODWT transform is similar to the discrete wavelet transform (DWT), but it does not produce a downsampling of wavelet coefficients [37], which allows it to overcome the lack of translation-invariance present in DWT and does not require the length of the signal to be a power of two. In contrast to CWT, MODWT calculates coefficients at scales 2*^j^* (where *j* is a level of transform) without the loss of information. This property provides faster computation of MODWT coefficients than CWT computation. The procedure of MODWT coefficient calculation can be described as an application of linear filters (wavelet and scaling filters) via a cascade algorithm (see Figure 3).

**Figure 4.**
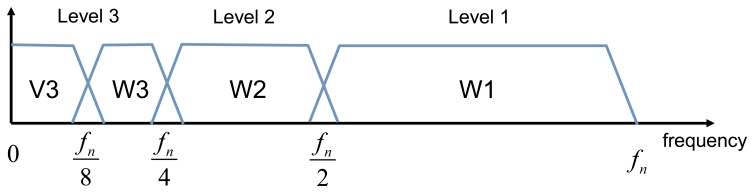
Frequency domain representation of MODWT levels. *f_n_*—upper limit in the frequency range of the analysed signal.

Frequency bands of MODWT levels are illustrated in Figure 4.

It is easier to work with the equivalent MODWT filters, which are analogous to wavelet and scaling functions, since the MODWT filters calculate coefficients by convolution with a signal directly [37]:

(1) $$V_{j,t}=\sum_{l=0}^{L_{j}-1}g_{j,l}\cdot X_{t-l}$$

where *X* is an analyzed signal; *j* is a level of decomposition; *V_j,t_* is a vector of approximation coefficients at level *j; g_j_* is a MODWT equivalent scaling filter; *L_j_* is defined by Equation (3).

(2) $$W_{j,t}=\sum_{l=0}^{L_{j}-1}h_{j,l}\cdot X_{t-l}$$

where *X* is an analyzed signal; *j* is a level of decomposition; *W_j,t_* is a vector of wavelet coefficients at level *j; h_j_* is a MODWT equivalent wavelet filter; *L_j_* is defined by Equation (3).

The length *L* of equivalent MODWT filters for the level *j* can be calculated using the following equation:

(3) $$L_{j}=\left(2^{j}-1\right)\left(L-1\right)+1$$

**Figure 5.**
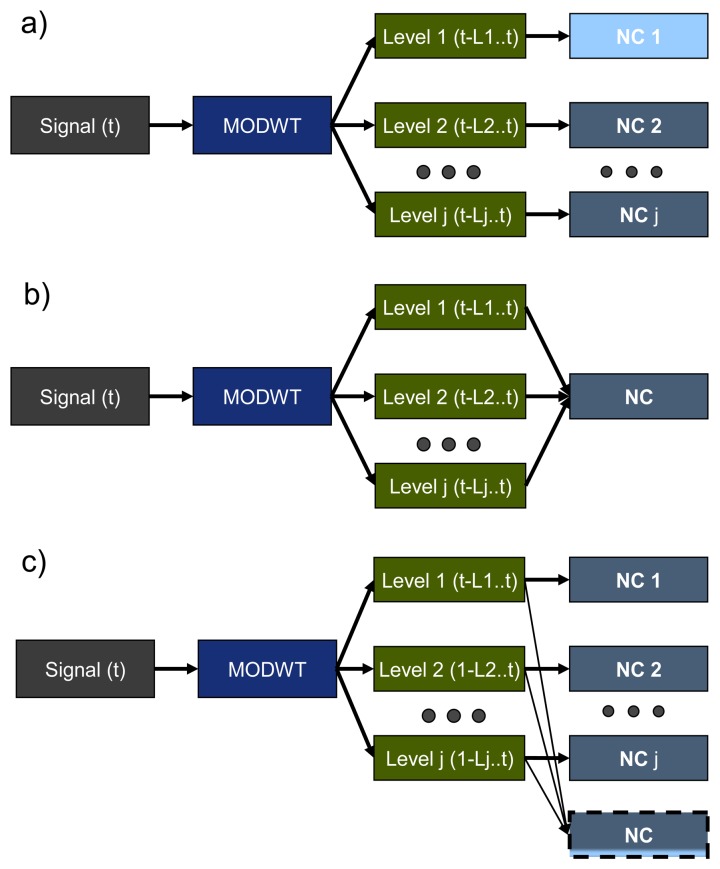
Three options for Maximum Overlap Discrete Wavelet Transform (MODWT) combination with Neural Clouds (NC). (**a**) Each level of MODWT decomposition is analysed by the individual instance of NC. (**b**) All levels of MODWT decomposition are analysed by one instance of NC. (**c**) A combination of (a) and (b).

MODWT and a one-side classifier (the Neural Clouds (NC) method is described later in this Section) can be combined in different ways (see Figure 5): each level of decomposition can be checked by individual NC (Figure 5a); or all levels can be grouped for analysis by only one NC (Figure 5b); or a combination of both (Figure 5c). We used the second approach in our work.

#### 2.2.2. Phase Shift Approach

Another time-frequency feature selected for levee behaviour monitoring is the phase difference between oscillating signals of different sensors. This feature can be extracted from any monitored system with some periodic behaviour, for instance from electrical signals, vibrations or more complex engineering, microeconomic and socio-dynamic systems. A sudden change in frequencies, amplitudes or phase shifts indicates a potential problem in the system.

**Figure 6.**
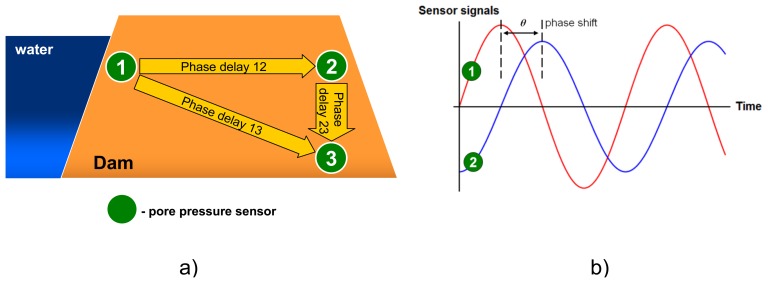
Levee health monitoring based on pairwise phase shift monitoring. (**a**) A scheme of the monitored levee (filled with permeable soil) and sensor positions. (**b**) Illustration of the phase shift concept: pressure wave caused by the sea tides is reaching sensor #2 with a time delay compared to sensor #1 located close to the river. This time lag is called “phase shift” or “phase delay” in signal analysis.

In case of levee health monitoring, tidal changes in water levels propagate through the soil inside the dike and cause periodic rises of water pressure at the sensors (see Figure 6). Since soil that fills the levee is a porous material with a relatively low permeability, water flow experiences resistance and reaches the sensors with some delay: the further from the sea the longer the time delay (called “phase shift” in signal analysis).

If the levee is stable (*i.e.*, the structure is not damaged and soil layers are not eroded), then the “resistivity” of porous levee remains constant, and consequently the phase shift between the sensors stays constant, too. A change in the phase difference will show that the levee integrity might be corrupted. Moreover, it will also point at the exact location of a problem—between the two sensors that revealed an anomalous phase shift.

Similar to the water pressure dynamics, other sensors also respond to the dynamically changing hydraulic forces caused by the tides, therefore our methodology can be applied also to sensors measuring inclination, displacement, stress, strain, and other levee health parameters.

A more detailed description of the phase shift approach and its applicability in early warning systems can be found in [38], where a finite element analysis and analytical solutions have been compared to the sensor data from the “Livedike” levee located in Groningen (The Netherlands).

We consider a phase shift between the Fourier transform components calculated for a selected frequency as the time delay metric.

Short-Time Fourier transform (STFT) is the right method that represents the signal in both time and frequency domains [39]. This property facilitates detection of anomalies by tracking the phase changes over time.

Time-frequency representation by STFT is performed by a discrete Fast Fourier Transform (FFT) algorithm in a sliding window. Each new sliding window overlaps with a previous window in order to reduce the boundary effects. STFT coefficients have a time delay at each frequency. STFT for discrete time series is given by:

(4) $$X(n,\omega )=\sum_{m=-\infty }^{\infty }x\left[m\right]w\left[n-m\right]e^{-j\omega n}$$

where *x*[*m*] is the analysed signal; *w* is the window function (e.g., Rectangular window, Hamming, Gaussian [39]); *x*[*m*]*w*[*n*−*m*] is the short-time section of the signal *x*[*m*] at time *n*.

Phase *ϕ* at timestamp *n* of frequency *ω* can be calculated as an argument of each STFT component [40]:

(5) ϕ(n,ω)=arg(X(n,ω))

Phase delay *τ_ϕ_* is then calculated as:

(6) $$\tau_{\phi }=\frac{\phi (n,\omega )}{\omega }$$

The minus in the formula (6) is required for the positive presentation of the delay in time domain.

Phase shift Δ*ϕ* (in radians) and time delay Δ *τ_ϕ_*(*n*, *ω)* in seconds between two selected sensors is calculated as:

(7) $$\Delta \phi \left(n,\omega \right)=\phi_{1}(n,\omega )-\phi_{2}(n,\omega )$$

(8) $$\Delta \tau_{\phi }(n,\omega )=-\frac{\Delta \phi (n,\omega )}{\omega }$$

where *ϕ*_1_( *n*, *ω*) is the phase of sensor #1; *ϕ*_2_( *n*, *ω*) is the phase of sensor #2.

A pairwise phase shift analysis can be extended to the analysis for sensor triplets (Figure 6a). Phase delay within the triangle should not change in time. Application of each individual sensor in phase shift analysis of several pairs guarantees redundancy. This means that detection as well as localization of the anomaly is possible. A phase delay is calculated from the time-frequency components related to two sensors, therefore we classify this feature as a ‘spatial time-frequency feature’.

**Figure 7.**
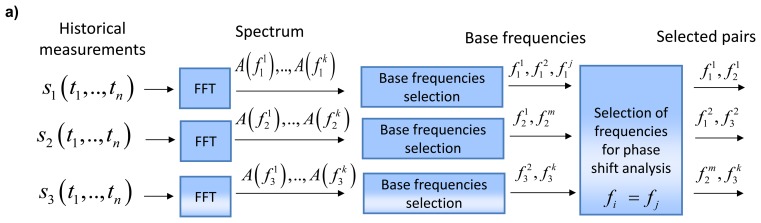

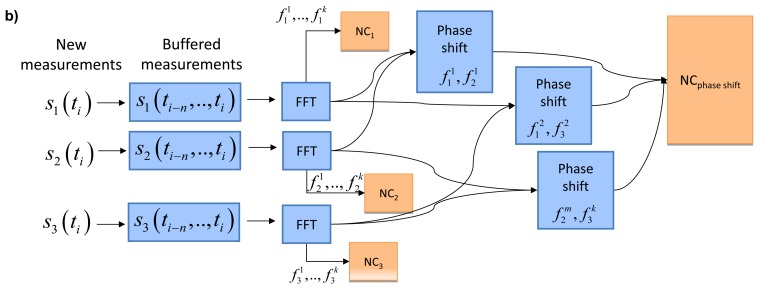
(**a**) Selection of pairs of FFT components for phase delay analysis in off-line mode. (**b**) On-line procedure of condition monitoring based on phase delay analysis. *s_j_*(*t*_1_,…*t_n_*) are measurements of the *j*-th sensor for time interval [*t*_1_,*t_n_*], $A\left(f_{j}^{i}\right)$ is the amplitude of FFT component of the *j*-th sensor for the *i*-th frequency.

A data analysis workflow is clarified in Figure 7. The fundamental (base) frequencies are selected (Figure 7a) after calculation of the spectrum [41] during the off-line stage. If two sensors have the same base frequencies, this pair is selected for further phase delay analysis. For example, three pairs are selected in Figure 7a. On-line monitoring (Figure 7b) presumes that one-side classifiers (marked as “NC” - Neural Clouds that are described later in this Section) are trained on FFT features that are extracted for the same base frequencies.

Spectra are analysed by individual instances of one-side classifiers (“NC_1_”, “NC_2_”). Calculated phase shifts (delays) for the selected on the off-line stage pairs of sensors are analysed by the separated instance of one-side classifier (“NC _phase shift_”). In most monitored systems, including levees, sensors measuring the same physical parameter have similar spectra. In our case of the semi-diurnal sea tides, one fundamental frequency is observed in all levee measurements: approximately 12 h, thus we can use the same base components for phase shift analysis.

### 2.3. Neural Clouds

The Neural Clouds (NC) classification algorithm receives pre-processed data and a set of extracted features as an input. The core of the NC classification agent (a single classification algorithm) is a combination of an Advanced K-Means (AKM) clustering algorithm and an extended radial basis functions network approach [25].

**Figure 8.**
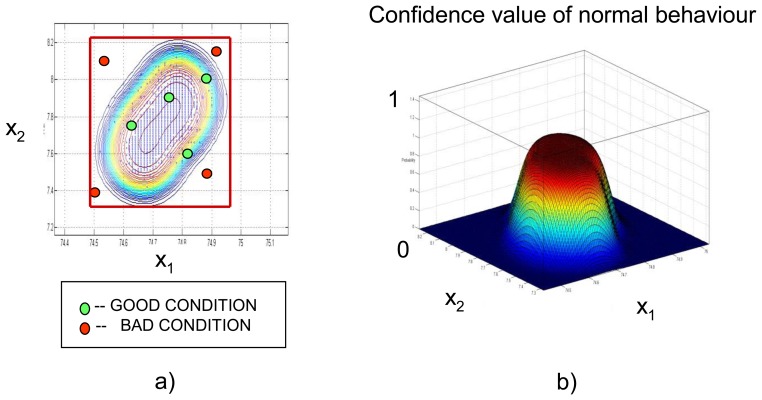
(**a**) Example of the Neural Clouds application to 2-D data. (**b**) 3-D presentation of the confidence values: value close to 1 is related to a normal behaviour, values close 0 can be interpreted as anomalies.

The NC encapsulates in a hypersurface all previously known configurations of selected parameters for a given training period (Figure 8a). It provides a more accurate classification of multidimensional data in normal and abnormal conditions in comparison to a simple hypercube approach. Red points in Figure 8a are incorrectly classified by the hypercube approach as ‘normal’ mode and are correctly classified by NC to reflect ‘abnormal’ mode. After training, the NC calculates a confidence value for every new state of the dike. Figure 8b shows a 3D presentation of the NC: X-Y plane that contains 2D data shown in a, the Z-axis interprets behaviour of the monitored object: values close to 1 are related to normal behaviour, values close to 0 can be interpreted as anomalies.

Each one-side classification instance can use either raw data or the features extracted from data, e.g., mean value of the signal calculated within a sliding window. Time-frequency methods for data pre-processing were previously studied in the *UrbanFlood* project in work [27], where Short Time Fourier Transform was used for pre-processing the Zeeland (in The Netherlands) levee data.

### 2.4. Implementation

The proposed anomaly detection approach is implemented within the AI component [42] of the *UrbanFlood* early warning system. This component architecture utilizes a cloud computing infrastructure of the EWS. Each AI component instance is a separate Virtual Machine (VM) working on a virtualization host. Any required number of AI components can be started and configured. More details can be found in [22].

## 3. Description of the Sensor Technologies Used in the *UrbanFlood* Project

### 3.1. AlertSolutions

**Figure 9.**
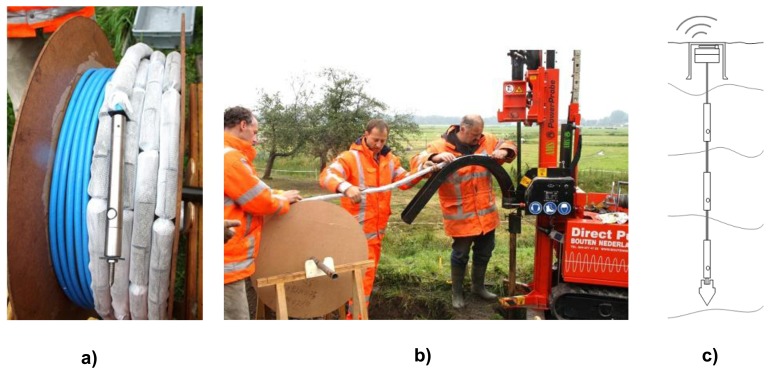
(**a**) GeoBeads sensor string with integrated bentonite shells for hydraulic isolation between measurement depths. (**b**) Installation of a GeoBeads string using a light weight direct push-in rig with sonic drilling head. (**c**) Schematic overview of a GeoBeads sensor string installed vertically for multilevel measurements as applied in levees, mountain slopes and construction activities.

In 2008 Alert Solutions introduced its sensor network GeoBeads for levee and slope stability monitoring. GeoBeads was developed to provide all essential dynamic parameters for the determination of soil stability (Figure 9a). GeoBeads consist of fully digital sensor modules (nodes) based on robust semiconductor technology that can scale to a wide area network of measurement locations. Each small sized node in the network can simultaneously house various measurement devices. The set per node commonly includes a piezometer, an inclinometer and thermal sensors [43,44]. Local contractors execute the installation with regular drilling or push-rig equipment (Figure 9b). Data is immediately available and is distributed via the internet.

### 3.2. GTC Kappelmeyer

**Figure 10.**
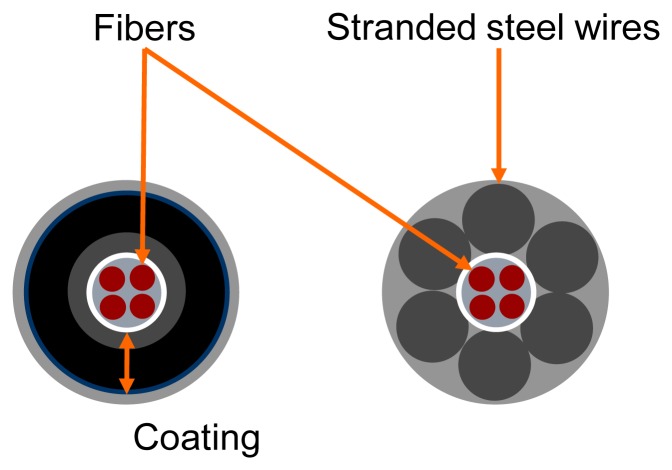
Cable structure and measuring principle. Schematic display of different cable types.

GTC Kappelmeyer uses the technology of distributed fibre optic temperature sensing that offers the possibility to measure the ambient temperature along fibre optical cables with high accuracy. This measuring method is based on the fact that the optical properties of the fibre depend on the ambient temperature. Fibre optical cables suitable for applications in hydraulic engineering usually consist of a core with at least two fibres and of a mechanical strength member covered by an outer jacket (Figure 10).

Distributed fibre optical temperature measurements are because of their high information density most suitable for levee seepage detection [45]. There are basically two measurement methods [46]: gradient method and heat-pulse method.

The first method requires temperature difference between the reservoir water and the dam material for successful leakage detection. The leakage is detected by a significant drop of the temperature gradient between the water and the ground temperature.

**Figure 11.**
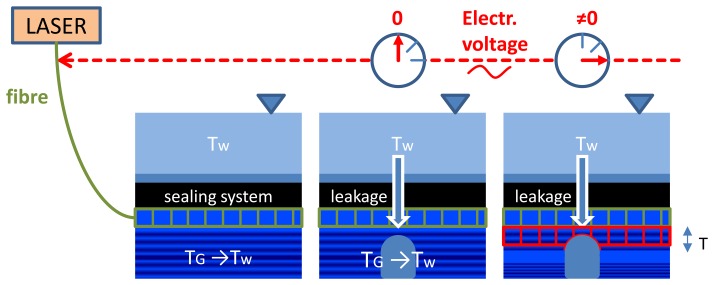
Heat-pulse method of leakage detection [48].

GTC Kappelmeyer uses a heat-pulse method [47] that provides robust leakage detection also in autumn and spring when ground (T_G_) and water (T_W_) temperatures are nearly equal (Figure 11). Area of leakage is presented with an arrow in the central area of central and right pictures in Figure 11. The fibre optic cable is heated up (central picture in Figure 11) by sending an electrical current through the metallic cable coating (e.g., rodent protection, shielding). The temperature increase in the immediate surroundings of the cable depends on the heat capacity and heat conductivity of the outcrop. While having seepage flow the poorly conductive heat transport is superimposed by the much more effective advective heat transport. Therefore, clearly visible temperature anomalies arise in these areas during the process of heating (difference of temperatures is shown with red figures in the right hand picture in Figure 11): temperature in area of seepage is lower in comparison to dry soil.

## 4. Results of Anomaly Detection in Three Operational Levees

### 4.1. Rhine Levee Data Analysis

#### 4.1.1. Rhine Levee Sensor Installation

**Figure 12.**
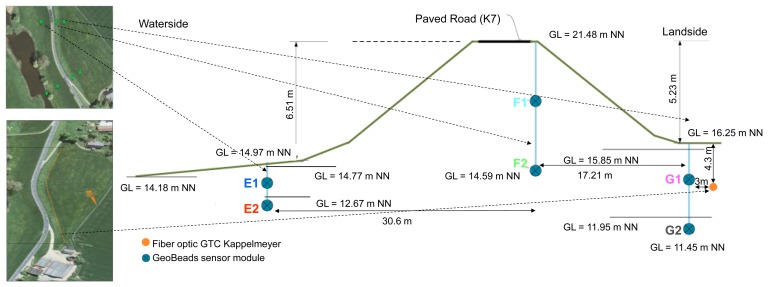
Alert Solutions sensors (marked with green balloons—left part of the picture, marked with blue circles—right part of the picture) and GTC Kappelmeyer fibre optic cable (marked with orange line) installed into the Rhine levee—second cross-section, left slope—waterside slope of the levee, right—landside slope.

There are two types of sensors installed in the Rhine river levee: Alert Solutions (GeoBeads) in 2 cross-sections and a GTC Kappelmeyer fibre optic cable (250 m) placed across the levee (Figure 12). One of the two Alert Solutions cross-sections is presented in Figure 12.

**Figure 13.**
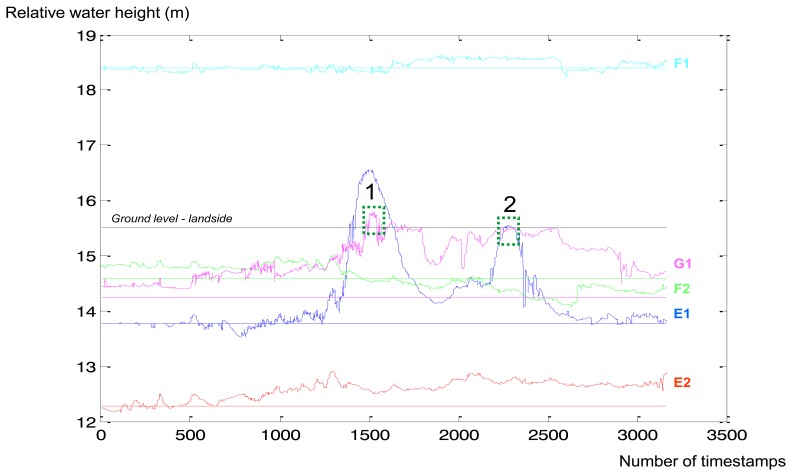
Alert Solutions pore pressure measurements converted to water level [m] in relation to Normaal Amsterdams Peil (NAP) or Amsterdam Ordnance Datum (normal water level in the Netherlands)—Y axis. X axis: number of timestamps, rate—1 h (period: November 2011–February 2012). Positions of sensors plotted in Figure 12.

Placement of the GTC Kappelmeyer fibre optic is marked in Figure 12 in orange. The fibre optics provide spatio-temporal temperature measurements across the levee. Pore pressure measurements gathered from Alert Solutions sensors are converted to water level values (Figure 13). Each colour corresponds to a specific sensor (for example: the “E2” sensor is marked with red in Figures 12 and 13). There are two lines per sensor: a straight horizontal line indicates the depth of the sensor installation, curve lines show the water head above this sensor depth level. If the water level is above a straight line, then this sensor is “covered” with water.

The two green dotted boxes in Figure 13a indicate the dates when the water level was higher than the ground water level (G1 sensor): 1st box—9th of January 2012, 2nd box—25th of January 2012. These peaks correspond to peaks in the Rhine water level of 820 cm and 710 cm [47]. According to the reported data the levee was wet, but “strong seepage flow (>10^−4^ m/s) can be excluded.” Summarizing all the aspects mentioned above, Alert Solutions sensors are useful for levee behaviour classifications (dry/wet). If piping starts close to the GeoBeads sensors, it will be easily detected. GTC Kappelmeyer fibre optic sensor should be applied for leakage testing between cross-sections.

#### 4.1.2. Analysis of Sensor Data and Anomaly Detection

**Figure 14.**
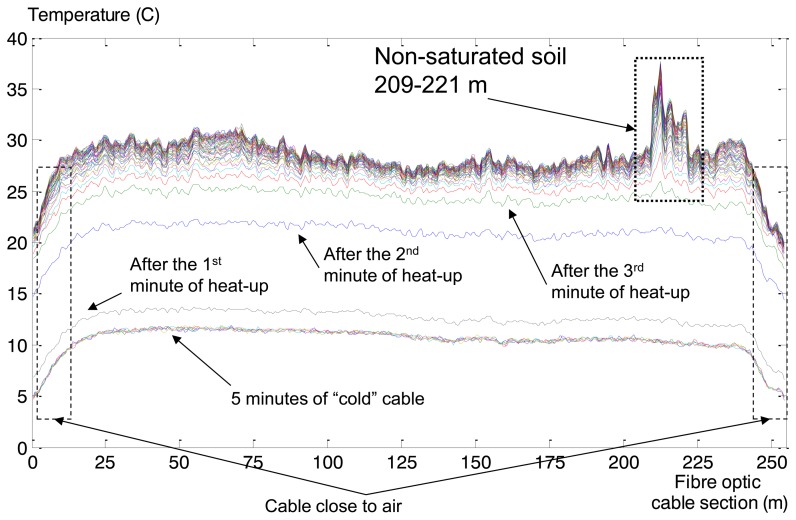
Rhine levee GTC Kappelmeyer fibre optic cable heat-up in time: X axis—length of the cable (m), Y axis—temperature (Celsius). The colours indicate different timestamps.

In this section we describe the collected GTC Kappelmeyer temperature measurements and the associated analysis. The process of a fibre optic heat-up is presented in Figure 14. As previously mentioned [47], there was no piping detected, so the levee's condition is normal. The only phenomena in collected temperature measurements that could be interpreted as anomaly is about 15 m wide area (Figure 14—dotted box) of non-saturated soil. This means that soil contains holes that contain air. Air has high insulation quality (thermal conductivity ∼0.4 W/m/K), which leads to measured high temperatures: the temperature across the whole cable is more or less the same during the heat-up process, about 28–30 degrees, but for the range of 209–221 meter (fibre cable optic length) the temperature is much higher.

**Figure 15.**
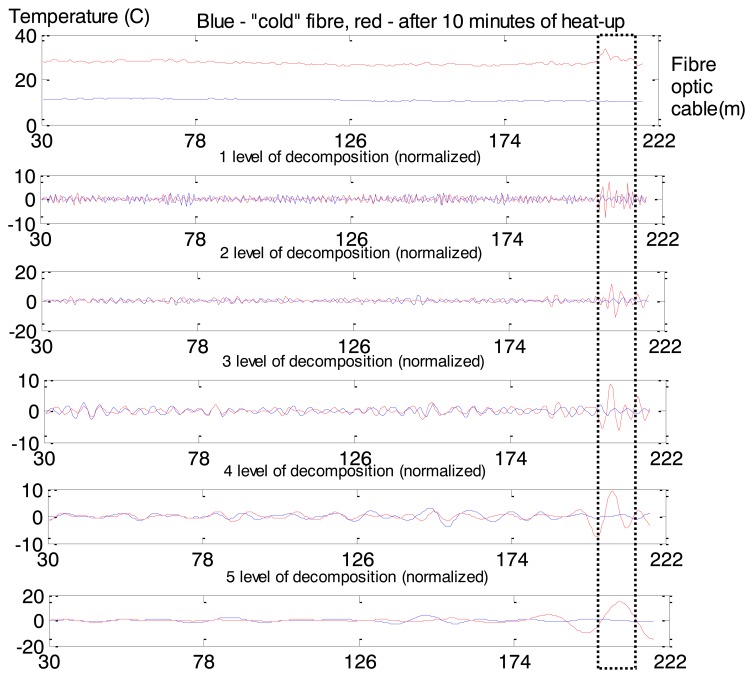
Results of MODWT application: X axis is the length of the cable (m), Y axis is temperature (Celsius). Dotted box shows position of the non-saturated part of the Rhine levee.

This effect can be interpreted as an anomaly and task of anomaly detection can be formulated as task of non-saturated soil detection. The Maximum Overlap Discrete Wavelet Transform (MODWT) was used for Rhine levee data analysis. Eight levels of decomposition (“la8”: least asymmetric wavelet with 8 levels of decomposition) are presented in Figure 15. As we can see from Figure 15 coefficients corresponding to the third and fourth levels of decomposition show the most protruding features. Therefore we apply them after post-processing as input for the one-side classifier. Measurements related to the cold fibre and several first minutes of heat-up were used for the NC training.

**Figure 16.**
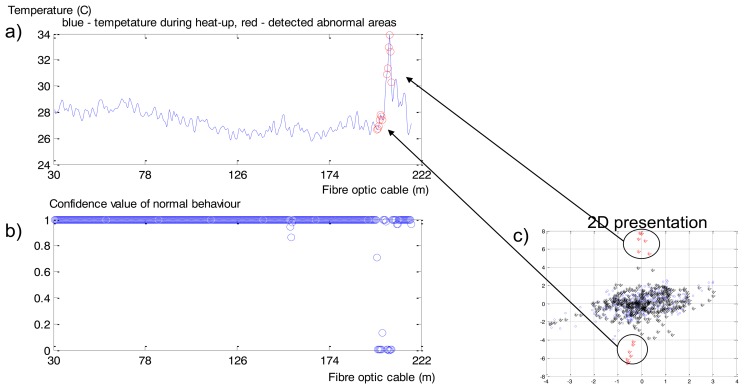
Non-saturated part of the Rhine levee detection: 10th minute of heat-up. (**a**) Input time series. (**b**) The calculated confidence values: values close to 1 are related to normal behaviour, close to 0 indicates anomaly. (**c**) The 3rd (X axis) and 4th (Y axis; levels of decomposition after post-processing: blue—training set, black—test set related to normal behaviour, red—test set related to anomaly.

Values in the spatial time series after 10 min of heat-up (Figure 16a) are presented as normal or abnormal points in Figure 16b: values close to 1 correspond to normal behaviour; close to 0 is interpreted as anomalies. Figure 16a is classified in Figure 16c by a 2D view of the constructed Neural Clouds. A cluster with normal data related to the training set is presented in Figure 16c with blue points, the test set is presented with black points and the detected outliers are marked in red.

MODWT does not have ‘perfect’ localization properties according to the Gibbs phenomenon [37], that is why some points are not correctly classified as abnormal behaviour: lower points in Figure 16c correspond to normal temperatures in Figure 16a. This example is a proof of principle of the functionality of the developed anomaly detection method. Application of this method to real leakage detection is presented in the next section.

### 4.2. Retaining Dam Data Analysis

**Figure 17.**
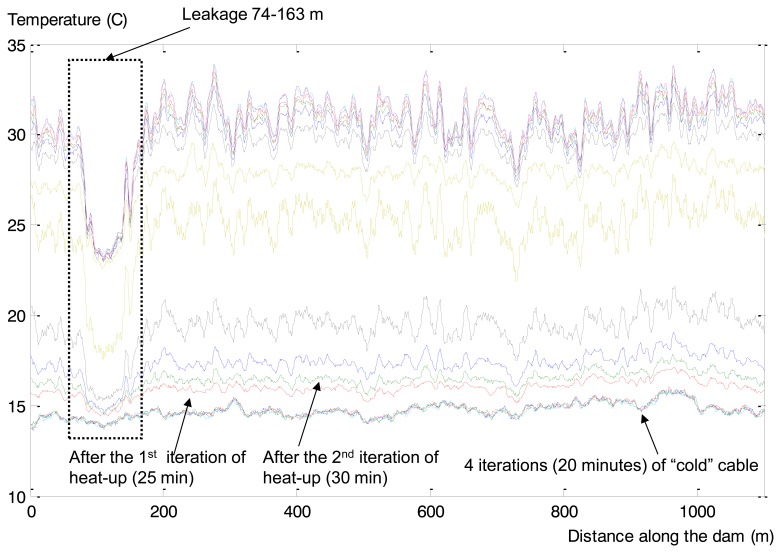
Retaining dam GTC Kappelmeyer fibre optic cable heat-up in time: X axis—length of the cable (m), Y axis—temperature (Celsius). The colours indicate different timestamps.

GTC Kappelmeyer provided for further testing of the developed anomaly detection method a real example of dam leakage, registered in measurements collected at an earth filled dam with bitumen sealing (total length more than 2 km). It contained a leakage of a bitumen sealing made of asphalt-coated gravel and bitumen binder of the dam. This anomaly is presented in spatial time series as a temperature drop in the range of 74–163 m (Figure 17).

**Figure 18.**
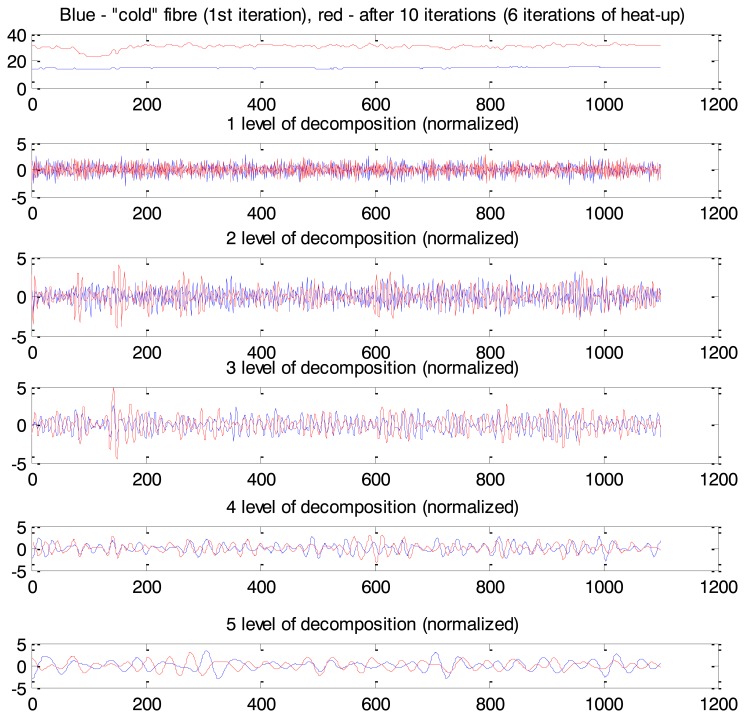
Results of MODWT application: X axis—length of the cable (m), Y axis—temperature (Celsius).

MODWT has been applied for spatio-temporal time series pre-processing (“la8”, eight levels of decomposition). The calculated wavelet coefficients are presented in Figure 18. Coefficients corresponding to the second and third levels of decomposition after post-processing were used as an input for the one-side classifier.

**Figure 19.**
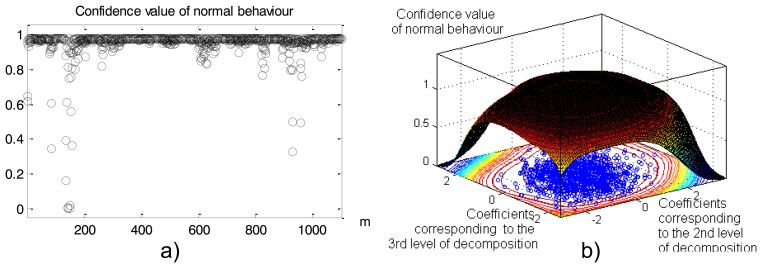
Retaining dam leakage detection: 6th iteration of heat-up. (**a**) The calculated confidence values: values close to 1 are related to normal behaviour, close to 0 mean anomaly. (**b**) 3D view of the constructed Neural Clouds.

Calculated confidence intervals (Figure 19a) show that the part of the cable between 150 m and 160m is classified as abnormal: confidence values are close to 0. A 3D presentation of the constructed NC is provided in Figure 19b. As we see the accuracy of the method is acceptable.

### 4.3. Boston Levee Data Analysis

#### 4.3.1. Boston Levee Sensor Installation

**Figure 20.**
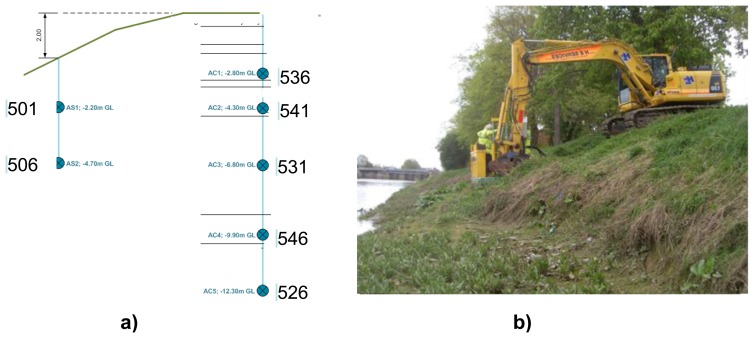
(**a**) One of two cross-section of the Boston levee, circles with numbers show installed pore pressure sensors. (**b**) A photo of the Boston levee.

The Boston dike (Figure 20) is one of the pilot sites of the *UrbanFlood* project. Detailed description of sensor networks installed into this dike can be found in [49]. The Boston location was of interest because there is a significant tidal forcing (up to six metres on spring tides). To monitor this dike successfully it is therefore important to monitor pore pressure, deformations, and ground water flow near the toe. Inclination measurements from the GeoBeads should provide an early clear sign of deformations, whilst differences in temperature measurement curves should be a strong indicator of water leakage [24].

The instrumentation and control building for the Grand Sluice [24] provided an ideal location for situating the computer equipment. Cross-section of this dike is shown in Figure 20a. The process of sensors installation is presented in Figure 20b.

#### 4.3.2. Analysis of Sensor Data and Anomaly Detection

**Figure 21.**
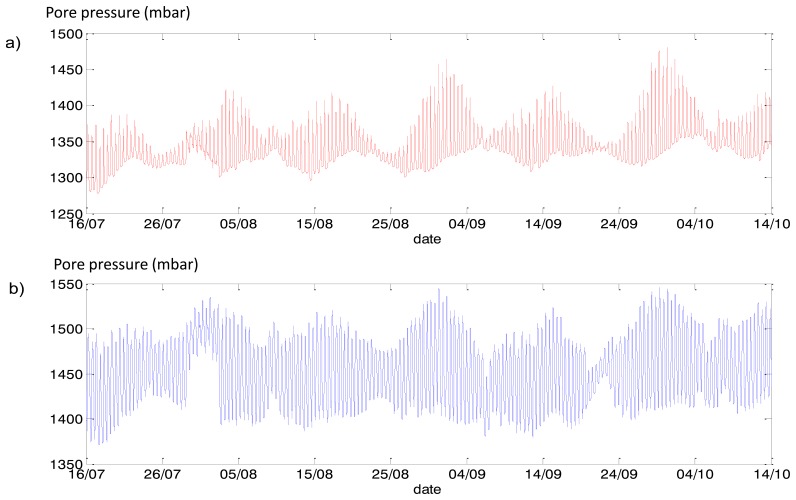
Pore pressure sensor data (**a**) #506, (**b**) #546.

We analysed all sensors, two (#506 and #546—see Figure 20a) were selected for presentation of phase delay analysis. Measurements of these sensors are presented in Figure 21.

**Figure 22.**
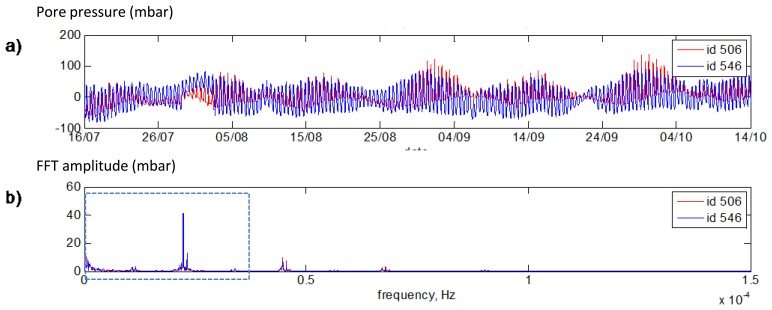
(**a**) Pore pressure data #506 (red line) and #546 (blue line) measurements after normalization (mean value was subtracted). (**b**) FFT spectra of #506 (red line) and #546 (blue line).

**Figure 23.**
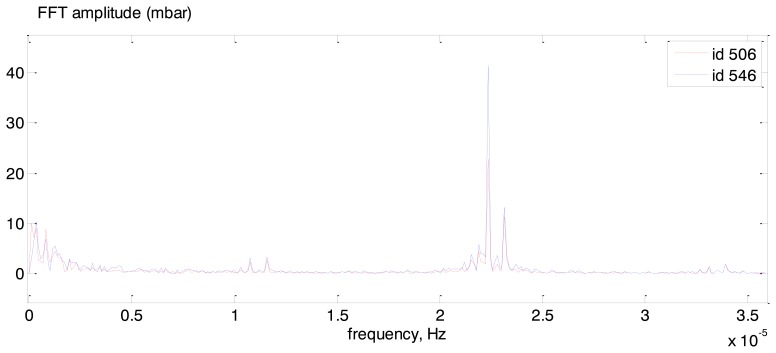
FFT spectra of #506 (red line) and #546 (blue line): zoomed in part of the Figure 22b.

Figure 22a presents measurements of both sensors after the mean value subtraction that is required for FFT application in order not to analyze the zero frequency. FFT spectra of both sensors are shown in Figure 22b. One can see that the set of base frequencies for both sensors is the same. The zoomed in part of the Figure 22b (area of blue dotted rectangular) is presented in Figure 23.

**Figure 24.**
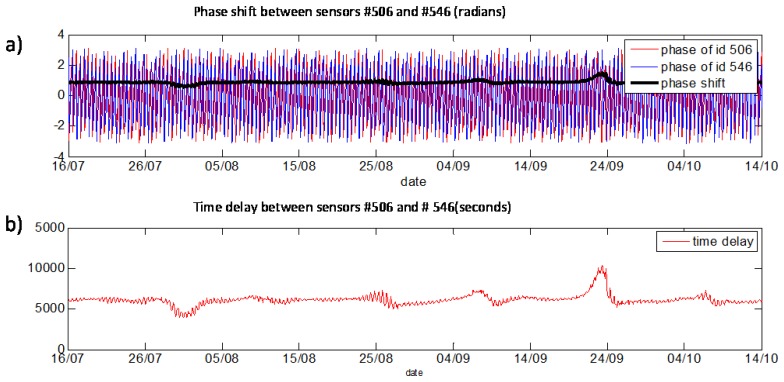
(**a**) Phase shift between two sensors for selected base frequency (12 h) (in radian): red line—phase of AS2 (id 506), blue line—phase of AC4 (id 546), black line—difference between phases. (**b**) Time delay between two sensors for selected base frequency (12 h) (in seconds).

The phase shift and time delay between both signals were analyzed for the same base frequency (12 h) extracted from the FFT spectra. In Figure 24b one can see the phases of #506 (blue line), #546 (red line) and difference between two phases (black line) calculated with a sliding window (1,024 timestamps, shift for 32 timestamps). Presentation of the time delay can be found in Figure 24c. It can be concluded that the time delay between sensors is stable (approximately 1 h and 40 min). For high/low tide cycles with frequency of 12 h the #546 sensor shows similar behaviour in comparison to #506 sensor with a delay of 1 h and 40 min. Preliminary results show that stability of phase shift between sensors can be used for the whole dike stability evaluation.

**Figure 25.**
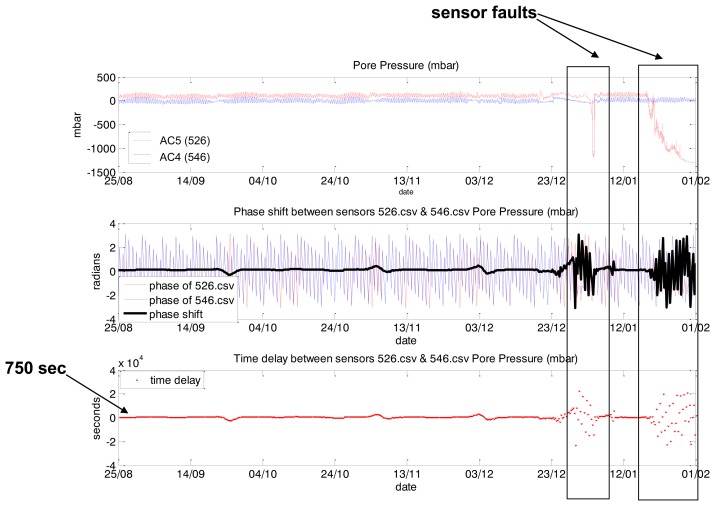
Application of the phase delay method to pore pressure sensors analysis: AC5 (id 526) with periods of abnormal behaviour and “normal” AC4 (id 546) from Boston dike (UK). Phase delay was calculated for one of the base frequencies—12 h, width of window for STFT calculation—512 samples, rate of measurements—15 min.

Identification of a sensor fault is presented in Figure 25. Two sensors were considered: #526 and #546. The first one started to show abnormal behaviour since end of December 2011.

For periods of #526 abnormal behaviour phase delay showed high deviation from phase delay calculated for “normal” behaviour of both sensors. This means that this feature can be used as input for further one-side classification for object's behaviour evaluation.

## 5. Conclusions

Anomalies can be a sign of a developing failure of a monitored object, so identification of an anomaly is an important step in structural health monitoring (SHM). In this paper we consider flood defence structures (earthen and concrete dams) as the object of monitoring. A combination of signal processing methods is presented for anomaly detection in measurements gathered from sensor networks installed into *UrbanFlood* earthen dams (a Rhine levee in Germany and the Boston levee in the UK) and in a retaining dam (in Germany).

There are two types of sensor networks installed into the levees that were considered in this paper: Alert Solutions and GTC Kappelmeyer. Alert Solutions sensors (GeoBeads) installed in the Rhine and Boston levees measure pore pressure, inclination and temperature in one point simultaneously. The GTC Kappelmeyer fibre optic cable installed in the Rhine levee and retaining dam measures temperature following the so-called active principle where the cable is heated [50].

Two types of anomalies were considered in this work. The first is a faulty abnormal behaviour. It was presented as a non-saturated soil in fibre optic measurements at the Rhine levee and as “strange” behaviour of some pore pressure sensors at the Boston levee. Both levees are not affected by leakage and no other anomalies were found. The third data set contains a pattern of real leakage (second type of anomalies) at the retaining dam.

All data sets were analyzed using an anomaly detection approach developed within the *UrbanFlood* project. A one-side classification approach (Neural Clouds [25]) in combination with a feature selection stage provides robust detection of anomalies. Neural Clouds do not require anomalies in a training set. Time-frequency methods were selected at the feature selection stage since they combine presentation of the input signal in both time and frequency domains: frequency analysis of the signal permits application of the anomaly detection approach to objects with different nature: slow processes (tides in case of earthen levees) and fast processes (vibrations in case of concrete structures); time presentation is often required for comparison of values with thresholds.

The anomalies in GTC Kappelmeyer measurements have been detected as a change in the spatial time series (values for some regions were much higher or lower in comparison to other sections of the cable). Maximum Overlap Discrete Wavelet Transform (MODWT) was selected as a feature selection procedure. This method is useful for detection of a change in the observed time series.

Analysis of phase delay between two sensors based on Fast Fourier Transform (FFT) applied in time provides robust identification of the sensor fault occurred at the Boston levee. This method analyses stability of dependencies between two sensors in the frequency domain.

There are some limitations of the phase delay approach. It only works when applied to periodic processes. In other case application of time-domain data-driven methods for transfer function modelling is more preferable, e.g., artificial neural networks and autoregressive model with exogenous inputs as previously reported in [28].

The developed anomaly detection approach can be applied for monitoring of various objects within SHM domain: it can be used for artificial (e.g., bridge, concrete dam, building) or natural (e.g., levee) constructions monitoring. The only requirements are installation of sensors providing the comprehensive information about important objects parameters; and availability of the on-line stream of data for on-line analysis.

Our anomaly detection approach is implemented within an Artificial Intelligence (AI) component that can be integrated in any existing decision support system. Condition monitoring using the *UrbanFlood* Early Warning System (EWS) provides a scalable solution: the required number of Virtual Machines (VM) containing the AI component can be initiated in the cloud computing infrastructure of the EWS on demand.

Automatic procedure for relevant feature selection is to be considered as one of the tasks for further research. Combining the proposed data driven approach in parallel with physical modelling, for instance the “Virtual Dike” ([11,22]) increases the robustness of the signal interpretation. Some preliminary studies along these lines were presented in [42].

## Notes

**Author Contribution:** Alexander Pyayt and Alexey Kozionov performed sensor data analysis. Ilya Mokhov, Bernhard Lang and Robert Meijer contributed to the anomaly detection concept. Bernhard Lang organised installation of sensors in the Rhine river levee. Valeria Krzhizhanovskaya and Peter Sloot contributed to the data-driven modelling and to writing this manuscript. All authors discussed the results and edited the paper
